# Nicotine Analogues in Oral Pouch Products and Associated Marketing Claims

**DOI:** 10.1001/jamanetworkopen.2025.54883

**Published:** 2026-01-22

**Authors:** Joseph R. Ciancio, Lakshmi Kumar, Kriti Rastogi, Meghan B. Moran, Lauren Czaplicki, Michelle K. Page, Hasan Jamil, Maciej L. Goniewicz, Tory R. Spindle

**Affiliations:** 1 Department of Psychiatry and Behavioral Sciences, Johns Hopkins University School of Medicine, Baltimore, Maryland; 2 Department of Health, Behavior, and Society, Johns Hopkins University School of Public Health, Baltimore, Maryland; 3 Department of Health Behavior, Roswell Park Comprehensive Cancer Center, Buffalo, New York

## Abstract

This case series study analyzed the contents of nicotine analoge pouch products available in the US and documented associated marketing claims.

## Introduction

The US Food and Drug Administration (FDA) regulates the manufacturing, marketing, and distribution of tobacco products, defined as products intended for human consumption containing nicotine from any source.^1^ This regulatory oversight is critical to ensure products are manufactured with adequate quality control and marketed without tactics that foster youth initiation or mislead consumers.

Products purported to contain nicotine analogues, including 6-methyl-nicotine (6MN) and nicotinamide, have recently emerged. These products, which closely resemble tobacco products, present a new regulatory challenge because nicotine analogues are not currently under FDA’s purview. However, nicotine analogues are chemically similar to nicotine and may possess similar harms (eg, abuse liability and toxic effects).^2,3,4^ This study tested nicotine analogue pouch products available in the US and systematically documented associated marketing claims. To our knowledge, US-based research to date has focused on nicotine analogue e-cigarettes predominantly,^5^ and the lone study on nicotine analogue pouches was conducted in the European Union.^6^

## Methods

This case series study assessed 15 pouches from 5 brands marketed as containing nicotine analogues purchased in April 2025 (Table 1). Gas chromatography–mass spectrometry quantified nicotine, 6MN, and nicotinamide concentrations (eAppendix 1 in Supplement 1). Marketing content from product packaging and brand websites was systematically evaluated using a detailed codebook and multicoder method (eAppendix 2 in Supplement 1).

**Table 1.** Nicotine and Nicotine Analogue Quantification in 15 Nicotine Analogue Oral Pouches

Brand name	Trademark analogue name	Flavor	Labeled analogue contentper pouch, mg	Detected (S)-6-methylnicotine per pouch, mean (SD), mg^a^	Detected nicotine per pouch, mean (SD), mg^b^	Detected nicotinamide per pouch, mg^c^	Label accuracy, %^d^
Sett	Ceretine	Wintergreen	6	0.61 (0.10)	0.04 (0.01)	Less than LLOQ	10.2
		Lemon	6	0.57 (0.03)	0.06 (0.01)	Less than LLOQ	9.5
		Wintergreen	3	0.31 (0.02)	0.05 (0.00)	Less than LLOQ	10.3
		Lemon	3	0.31 (0.01)	0.05 (0.00)	Less than LLOQ	10.3
Str8up	Novatine	Wintergreen	12	1.98 (0.10)	Less than LLOQ	Less than LLOQ	16.5
		Citrus	12	1.83 (0.08)	Less than LLOQ	Less than LLOQ	15.3
Hippotine by Happy Hippo	Imotine	Wintergreen	25	1.05 (0.02)	Less than LLOQ	Less than LLOQ	4.2
		Guava	15	0.62 (0.03)	Less than LLOQ	Less than LLOQ	4.1
Upperdeckys by MG	Imotine	Wintergreen	15	0.60 (0.01)	Less than LLOQ	Less than LLOQ	4.0
		Buzzin’ Berry	8	0.32 (0.01)	Less than LLOQ	Less than LLOQ	4.0
		Orange Creamsicle	25	1.04 (0.03)	Less than LLOQ	Less than LLOQ	4.2
Outlaw^e^	Nixamide/Nic-Safe (nicotinamide)	Wintergreen	NA	0.13 (0.01)	Less than LLOQ	Less than LLOQ	NA
		Creamsicle	NA	0.11 (0.00)	Less than LLOQ	Less than LLOQ	NA
		Georgia Peach	NA	0.12 (0.01)	Less than LLOQ	Less than LLOQ	NA
		Backwoods Blueberry	NA	0.11 (0.00)	Less than LLOQ	Less than LLOQ	NA

Abbreviations: LLOQ, lower limit of quantification; NA, not applicable.

^a^ The (S)-6-methylnicotine LLOQ is 0.1 mg per pouch.

^b^ The nicotine LLOQ is 0.02 mg per pouch.

^c^ The nicotinamide LLOQ is 0.1 mg per pouch.

^d^ Percentage of labeled analogue dose (per pouch) as stated on product labels, detected via chemical analysis of nicotine and nicotine analogue content.

^e^ Outlaw pouches did not list the analogue dose size on physical packaging or on their website; therefore, percent differences could not be calculated.

## Results

Products were labeled as containing 3 to 25 mg of distinct trademarked nicotine analogues. 6MN was detected in pouches at concentrations of 4.0% to 16.5% of the stated analogue dose on labels. Nicotinamide was not detected in any pouch, including Outlaw, which claimed to contain nicotinamide (Nic-Safe). Nicotine was detected at trace levels in all Sett pouches but no other products (Table 1).

Common marketing claims included pouches being nicotine free and superior to nicotine (eg, less addictive); 3 brands alleged to have supportive scientific evidence for these claims (Table 2). Four brands featured addiction risk warnings, and 3 claimed to be FDA exempt. The availability of flavors was often highlighted.

**Table 2.** Representative Marketing Content and Related Claims Featured on Nicotine Analogue Pouch Product Labels and Brand Websites^g^

Brand name	Addiction risk^a^	Provides a buzz^b^	Less addictive^c^	Nicotine free^d^	FDA exempt^e^	Toxicology testing^f^
Sett	Yes	Yes	Yes	Yes	Yes	Yes
Str8up	Yes	No	No	Yes	Yes	No
Happy Hippo	Yes	No	No	Yes	No	Yes
MG	Yes	Yes	No	No	No	No
Outlaw	No	Yes	Yes	Yes	Yes	Yes

Abbreviation: FDA, US Food and Drug Administration.

^a^ Warnings to the consumer.

^b^ General claims about the attributes or effects of the products.

^c^ Claims specifically comparing the nicotine analogue products to traditional nicotine products.

^d^ Specific key terms commonly featured on oral nicotine pouch products.

^e^ Mentions of the FDA, including exemption status or approval.

^f^ Claims of scientific testing, validation, and laboratory-based evidence.

^g^ Yes indicates marketing feature present; no indicates marketing feature not present. Product labels and websites were coded based on the presence of marketing claims and tactics across 6 categories (ie, safety warnings, general claims, nicotine comparison, pouch key terms, FDA claims, and scientific evidence claims). Two individuals performed the coding, and a third individual resolved any discrepancies. See eAppendix 2 in Supplement 1 for a full description of items coded.

## Discussion

This study highlights substantial concerns about nicotine analogue pouch products sold in the US. All products contained 6MN, a nicotine analogue with greater pharmacologic potency than nicotine^2,3,4^ and lacking clinical research to inform doses safe for human consumption.

All Sett pouches contained nicotine, contradicting claims of being nicotine free. This is the first study, to our knowledge, to detect nicotine in nicotine analogue products and underscores that this product, and possibly other untested analogue products, may be subject to FDA regulation if they contain detectable levels of nicotine. Notably, a European Union–based study similarly detected 6MN in various nicotine analogue pouches, though, unlike the current study, 6MN concentrations were similar to the labeled analogue dose and nicotine was not detected.^6^

Product labels and websites generally did not disclose the presence of 6MN, and products were instead marketed with ambiguous tradenames. Products also highlighted youth-appealing flavors and used tactics that may increase appeal, such as unsubstantiated claims of reduced harm compared with nicotine or being exempt from FDA regulation.

Concerningly, another brand (Outlaw) seemingly altered their nicotine analogue formulation without updating the product’s labeling. At the time of purchase (April 2025), Outlaw was marketed as containing nicotinamide, but only 6MN was detected (not nicotinamide). In August 2025, Outlaw’s website was updated to reflect that the product contains 6MN. Thus, our analysis suggests that this brand was mislabeled with the incorrect analogue for at least 4 months. Study limitations include a small convenience sample and a focus on only 2 nicotine analogues.

The availability of unregulated nicotine analogue pouches with addiction potential, misleading marketing claims, and inaccurate labeling is a public health concern that may undermine tobacco control efforts. Regulatory oversight of these products and more research to understand their potential harms are urgently needed to protect public health.

## Notes

Supplement 1. **eAppendix 1. **Gas Chromatography/Mass Spectrometry Methodology **eAppendix 2. **Nicotine Analog Oral Pouch Marketing Analysis Methods

Supplement 2.
Data Sharing Statement
