# Supplementary material for: Nicotine Analogues in Oral Pouch Products and Associated Marketing Claims
Source: JAMA Netw Open. 2026 Jan 22;9(1):e2554883. doi: 10.1001/jamanetworkopen.2025.54883 (PMC12828627; doi:10.1001/jamanetworkopen.2025.54883)
Supplement: Supplement 1. — eAppendix 1. Gas Chromatography/Mass Spectrometry Methodology eAppendix 2. Nicotine Analog Oral Pouch Marketing Analysis Methods [file jamanetwopen-e2554883-s001.pdf]

Supplemental Online Content

Ciancio JR, Kumar L, Rastogi K, et al. Nicotine analogues in oral pouch products and associated marketing claims. *JAMA Netw Open*. 2026;9(1):e2554883. doi:10.1001/jamanetworkopen.2025.54883

eAppendix 1. Gas Chromatography/Mass Spectrometry Methodology

eAppendix 2. Nicotine Analog Oral Pouch Marketing Analysis Methods

This supplemental material has been provided by the authors to give readers additional information about their work.

© 2025 Ciancio JR et al. *JAMA Network Open.*

eAppendix 1. Gas Chromatography/Mass Spectrometry Methodology

Table 1 – Method parameters for nicotine & nicotine analog assays

	Nicotine Assay	Analogs Assay
**Targeted Chemicals**	Nicotine	6-methylnicotine, Nicotinamide
**Purchased chemicals**	Nicotine: Fisher Scientific (neat)<br>Naphthalene-d8: Santa Cruz Biotechnology (neat)<br>LC/MS grade Methanol: Fisher Scientific	6MN: LGC (neat)<br>Nicotinamide: Fisher Scientific (neat)<br>Naphthalene-d8: Santa Cruz Biotechnology (neat)<br>LC/MS grade Methanol: Fisher Scientific
**Calibration range**	0.02-4.0 mg/pouch	0.1-8.0 mg/pouch
**Extraction Parameters**		
**Extraction Solvent & Internal Standard**	25µl/ml Naphthalene-d8 in methanol	25µl/ml Naphthalene-d8 in methanol
**Preparation Steps**	20 mL of methanol-based extraction solvent containing internal standard are mixed with one pre-weighed pouch for 15 min. Solutions are filtered through 0.45µm PFTE filter and analyzed following the analytical parameters, below. Each brand is analyzed in triplicate.	20 mL of methanol-based extraction solvent containing internal standard are mixed with one pre-weighed pouch for 15 min. Solutions are filtered through 0.45µm PFTE filter and analyzed following the analytical parameters, below. Each brand is analyzed in triplicate.
**Analytical Parameters**		
**Instrumentation**	Agilent 7890B/5977A GC/MS	Agilent 7890B/5977A GC/MS
**Column**	Agilent DB-624UI<br>(30m length, 0.32mm inner diameter, 1.8µm film thickness)	Agilent DB-624UI<br>(30m length, 0.32mm inner diameter, 1.8µm film thickness)
**Inlet**	EPC capillary split/splitless	EPC capillary split/splitless
**Injection Volume**	1µl	1µl
**Injection**	Splitless	Split 5:1
**Injection Port Liner**	Splitless; single taper, ultra inert liner with glass wool	Universal; low PSI drop, ultra inert liner with glass wool
**Inlet Temp**	250°C	250°C
**Septum Purge Flow**	3.0 ml/min	3.0 ml/min
**Gas Saver**	20 ml/min after 2.0 min	20 ml/min after 2.0 min
**Carrier Gas**	Helium, ramped flow	Helium, constant pressure
**Oven Program**	70°C for 0min, then 4°C/min to 80°C, hold for 1.0min, then 50°C/min to 180°C, hold for 0min, then 5°C/min to 240°C, hold for 0min, then 55°C/min to 260°C, hold for 9.2min	50°C for 2 min, then 10°C/min to 240°C, hold for 14.0min
**MSD Transfer Line**	250°C	250°C
**MSD Source/Quad Temps**	230°C / 150°C	230°C / 150°C
**MSD Acquisition Mode**	Scan, positive ionization	Scan, positive ionization
**Quantitation & Secondary ions**	Nicotine: 84, 133, 162	6MN: 84, 147, 175<br>Nicotinamide: 122, 78, 106

Note: Nicotine assay previously reported (DOI:10.1093/ntr/ntaf105)

© 2025 Ciancio JR et al. *JAMA Network Open.*

eAppendix 2. Nicotine Analog Oral Pouch Marketing Analysis Methods

Strategy for Identification of Products:

We identified nicotine analog pouches sold commercially in the US by searching Google using key terms including “non-nicotine pouches,” “nicotine free pouches,” and “Imotine,” “Ceretine,” “Novatine,” “Nic-Safe,” “nicotinamide” or “6MN.” Search terms were informed by prior studies focused on nicotine analog-containing e-cigarettes and by publicly available tobacco market surveillance alerts from the Rutgers University Center for Rapid Surveillance of Tobacco (CRST). Only products available via direct-to-consumer commercial sale in the US were included. We excluded products only accessible through wholesale distribution or manufactured internationally.

To evaluate the marketing content used by each of the selected brands, we systematically evaluated both the physical product packaging and websites. For physical products, we photographed all external sides of the tin (top, bottom, sides). For websites, we captured screenshots of key content areas such as home or landing pages, along with other relevant web pages describing the products (e.g., “about us” pages).

A code book was applied to both the physical product packing and each brand’s website at the time of capture. Importantly, this codebook was created *a priori* and was adapted from our ongoing research to document the marketing claims of oral nicotine pouches.

Table 2. Marketing Content on Physical Product Labels and Brands Websites

Code Book Item Number (see below)	Sett	Str8up	Happy Hippo	MG	Outlaw	% Brands
1.	Yes	Yes	Yes	Yes	No	80%
2.	Yes	Yes	Yes	Yes	No	80%
3.	Yes	No	Yes	No	Yes	60%
4.	Yes	No	No	No	Yes	40%
5.	No	No	No	No	Yes	20%
6.	No	No	No	No	Yes	20%
7.	No	No	No	No	No	0%
8.	No	No	No	No	No	0%
9.	No	No	No	No	Yes	20%
10.	No	No	No	No	No	0%
11.	Yes	No	No	Yes	Yes	60%
12.	Yes	No	No	No	No	20%
13.	Yes	Yes	Yes	No	No	60%
14.	Yes	Yes	Yes	Yes	Yes	100%

© 2025 Ciancio JR et al. *JAMA Network Open.*

15.	Yes	Yes	Yes	No	Yes	80%
16.	Yes	No	Yes	Yes	Yes	80%
17.	Yes	No	Yes	No	Yes	60%
18.	No	No	No	No	Yes	20%
19.	No	No	No	No	No	0%
20.	No	No	No	No	Yes	20%
21.	No	No	Yes	No	Yes	40%
22.	No	No	No	No	No	0%
23.	Yes	No	No	No	Yes	40%
24.	Yes	No	No	No	Yes	40%
25.	Yes	Yes	Yes	No	No	60%
26.	Yes	Yes	No	Yes	Yes	80%
27.	Yes	No	No	No	Yes	40%
28.	No	Yes	No	No	No	20%
29.	Yes	No	No	Yes	Yes	60%
30.	Yes	No	No	No	Yes	40%
31.	Yes	No	No	No	Yes	40%
32.	Yes	No	No	No	Yes	40%
33.	Yes	Yes	No	No	Yes	60%
34.	No	No	No	No	Yes	20%
35.	No	No	Yes	Yes	No	40%
36.	Yes	No	Yes	No	Yes	60%
37.	Yes	No	Yes	No	Yes	60%

© 2025 Ciancio JR et al. *JAMA Network Open.*

38.	Yes	No	Yes	No	No	40%
39.	No	No	No	No	No	0%
40.	Yes	No	Yes	No	No	40%

Note: Yes = marketing claim present; No = marketing claim not present.

The Code Book Item Number column correspond to each item included in the code book. A complete list of corresponding items is included in Appendix B. For all coded items, each brand is indicated if that item was present (yes or no) across both physical product packaging or brand websites.

Oral Nicotine-Analog Pouch Code Book Items:

Safety Warnings

1. **Age Restriction:** Are there statements of the product being restricted for sale 21+, including pop-ups that require age verification before entering the website, and any other age restriction warnings?
2. **Addiction Risk:** Are there statements regarding the potential addiction risk of the pouches, including website banners and warning notices on physical products?

General Oral Nicotine Pouch Claims

Does the physical product or website make claims which are commonly featured on other forms of oral nicotine pouches, not specific to their unique analog constituent. The product:

3. Is an alternative to other tobacco products
4. Is less harmful than cigarettes / e-cigarettes / dip chew
5. Is a tool to help quit smoking
6. Is convenient to use
7. Is affordable
8. Is discrete
9. Is mess-free
10. Is odor-free
11. Provides a "buzz"
12. Improves productivity and focus
13. Allows you to choose your preferred strength
14. Has a variety of high-quality flavors

Pouch Key Terms

Does the physical product or website use specific terms commonly featured on other forms of oral nicotine pouches? Items were only considered present if they appeared in the exact phrasing listed below.

15. Nicotine Free / Non-nicotine
16. Tobacco Free
17. Alternative
18. Pure
19. Simple
20. Smoke free
21. Spit free
22. Synthetic
23. Patented / Proprietary

Nicotine Comparison Claims

Does the physical product or website feature claims which specifically compare their analog containing product to traditional nicotine or tobacco. Compared to other forms of nicotine, the product:

© 2025 Ciancio JR et al. *JAMA Network Open.*

24. Provides the same experience without negative side effects (i.e. less toxic or harmful / safer)
25. Acts pharmacokinetically similar (references to receptor sites, brain activity, ect.)
26. Is structurally similar but distinct from nicotine
27. Is less addictive
28. Has a quicker drug delivery
29. Has a “smoother” drug delivery
30. Improves energy
31. Improves focus
32. Improves mood

FDA Claims

Does the physical product or website mention the FDA in any specific capacity as a means of marketing including explicit statements claiming products are FDA exempt, FDA approved, outside of FDA regulation or legal? The product is

33. FDA exempt or outside of FDA regulation
34. Approved by the FDA
35. Not currently evaluated by FDA (must be explicitly stated)

Scientific Evidence Claims

Does the physical product or website feature claims of scientific testing, validation, or lab-based evidence as a form of marketing?

36. The product is developed by scientists
37. Toxicology testing was conducted
38. Testing of pharmacokinetics was conducted
39. Testing of pharmacodynamic effects was conducted
40. Lab testing is mentioned but is unspecified / unclear

© 2025 Ciancio JR et al. *JAMA Network Open.*
