# Supplementary material for: Nicotine Analogues in Oral Pouch Products and Associated Marketing Claims
Source: JAMA Netw Open. 2026 Jan 22;9(1):e2554883. doi: 10.1001/jamanetworkopen.2025.54883 (PMC12828627; doi:10.1001/jamanetworkopen.2025.54883)
Supplement: Supplement 2. — Data Sharing Statement [file jamanetwopen-e2554883-s002.pdf]

Data Sharing Statement

Ciancio. Nicotine Analogues in Oral Pouch Products and Associated Marketing Claims. *JAMA Netw Open*. Published January 22, 2026. doi:10.1001/jamanetworkopen.2025.54883

Data
Data available: No

Additional Information
Explanation for why data not available: data will be shared upon reasonable request
